# Infliximab-Induced Non-specific Interstitial Pneumonitis in a Patient With Ulcerative Colitis (UC)

**DOI:** 10.7759/cureus.33064

**Published:** 2022-12-28

**Authors:** Syed Alishan Nasir, Ronak Patel, Steven Wojkiewicz, Robyn Scatena

**Affiliations:** 1 Internal Medicine, Norwalk Hospital, Norwalk, USA; 2 Radiology, Norwalk Hospital, Norwalk, USA; 3 Pulmonary and Critical Care Medicine, Norwalk Hospital, Norwalk, USA

**Keywords:** tnf alpha inhibitor, adverse drug effect, nonspecific interstitial pneumonitis, drug induced interstitial lung diseases, : infliximab

## Abstract

Infliximab belongs to the family of tumor necrosis factor (TNF) alpha inhibitors and since its development, it has revolutionized treatment for both rheumatological diseases and inflammatory bowel disease (IBD). In IBD specifically, it has shown to result in symptomatic, endoscopic, and histological remission which is why it is one of the most widely used treatments for moderate to severe IBD. While common side effects include infections due to immunosuppression, cytopenias and hepatotoxicity, interstitial lung disease (ILD) has been infrequently reported to result from inflixiamb use. We present the case of a patient with ulcerative colitis (UC) who achieved remission with infliximab, however, after about two years of infusions, developed evidence of non-specific interstitial pneumonitis (NSIP). Extensive work up was done to rule out infections, mixed connective tissue disorders, and hypersensitivity pneumonitis. Although lung biopsy remains the gold standard for diagnosing NSIP; clinical, laboratory, and radiographic findings were sufficient in establishing this diagnosis. Initiation of empiric high dose steroids, and cessation of infliximab infusions showed improvement in respiratory status and resolution of lung findings. This case highlights the importance of recognizing adverse effects of infliximab on the pulmonary status of an IBD patient given that infliximab mediated ILD does not adhere to a specific timeline. Considering that respiratory function may be compromised post any number of infusions, it is imperative to acknowledge patients' respiratory complaints and initiate prompt investigation and evaluation for this rare complication.

## Introduction

Infliximab is a monoclonal antibody that targets TNF alpha and is used for the treatment of moderate-severe active ulcerative colitis (UC). Common side effects include infections, cytopenias, and infusion reactions. Although rare, there have also been case reports describing lung toxicity resulting from infliximab therapy in the form of non-specific interstitial pneumonitis (NSIP) which is one class of idiopathic interstitial pneumonia (IIP) characterized by homogenous appearance of interstitial fibrosis and inflammation. NSIP can be idiopathic or associated with connective tissue disease, autoimmune diseases such as inflammatory bowel disease (IBD), HIV infections, toxins, and drugs. NSIP is rare and constitutes up to 14%-36% of IIP [1]. While a lung biopsy is the gold standard for diagnosis, this is often not required and a diagnosis can be established with a multidisciplinary approach based on history, laboratory findings, and imaging. Treatment of NSIP depends on the cause, for example treatment of underlying connective tissue disease, removal of drug or antiretroviral therapy in the case of HIV. Steroids can be used in the interim for symptom management [1]. Here we present a case of a patient diagnosed with UC and started on infliximab therapy for two years who presented with acute hypoxemic respiratory failure and was diagnosed with NSIP based on clinical, laboratory, and imaging findings. After withdrawal of infliximab followed by a steroid course, the patient was noted to have complete resolution of his lung findings based on imaging studies.

## Case presentation

**Figure 1:**
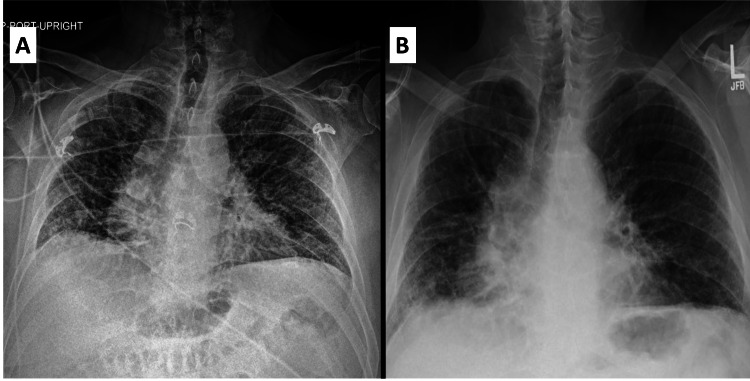
CXR showing evidence of interstitial lung disease and resolution. A, CXR on admission; B, CXR after 4 weeks of steroids CXR, chest X-ray

A 72-year-old male with a medical history of UC diagnosed at the age of 65 and on treatment with infliximab for the past two years, vaccinated against COVID with the primary series of vaccines, no prior history of lung disease or connective tissue disease presented to the hospital for worsening dyspnea on exertion refractory to antibiotic use. On initial evaluation the patient was afebrile, tachycardic, tachypneic, normotensive, and saturating at 84%-85% on room air. Laboratory studies were grossly unremarkable. Physical exam demonstrated diffuse crackles in all lung fields. Chest X-ray (Figure 1A) showed a low lung volume with worsening coarse reticular lung abnormalities indicating interstitial lung disease (ILD). 

**Figure 2:**
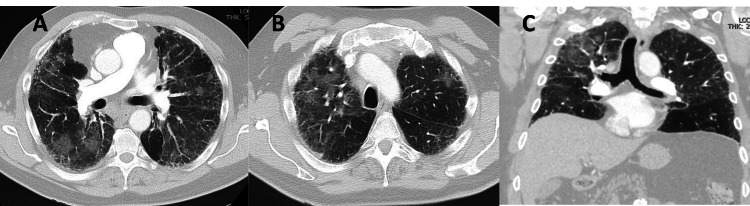
CTA showing ground glass opacities consistent with ILD. A, transverse section of lung (proximal); B, transverse section of lung (distal); C, sagittal section of lung CTA, CT angiograph; ILD, interstitial lung disease

Computed tomography angiograph (CTA) of the chest was obtained which showed evidence of airway-centric ILD characterized by peripherally predominant reticulations with patchy ground glass opacities, absence of honeycombing, evidence of bronchiectasis and absence of consolidations (Figure 2A-C). Together these findings were concerning for chronic hypersensitivity pneumonitis vs mixed-type NSIP.

A pulmonologist was consulted and initial suspicion was for infectious process including bacterial/viral pneumonia as well as fungal pathogens given immunocompromised status secondary to infliximab infusions. The patient was started on empiric antibiotic coverage with ceftriaxone and doxycycline, prior to which extensive infectious work-up was performed including blood cultures, legionella and streptococcus urine antigens, a respiratory viral panel (which included influenza A/B, respiratory syncytial virus, enterovirus, and rhinovirus) and fungal assays, all of which were negative. The patient’s infliximab (last infusion two weeks prior to admission) was also held due to concern for drug-induced ILD. Extensive rheumatological workup was also performed to look for underlying connective tissue disease but yielded negative results for antineutrophil cytoplasmic antibodies (ANCA), anticentromere antibodies, rheumatoid factor, anti-cyclic citrullinated peptide antibody, anti-SCL, anti-SSA, anti-SSB, anti-double-stranded DNA antibody, anti-Jo 1, and aldolase antibody. Serologies were positive for only anti-nuclear antibodies (ANA) with a titer greater than 1:2560. He underwent bronchoscopy within 36 h of admission which showed no evidence of diffuse alveolar hemorrhage and normal appearing lung lobes on inspection. Bacterial and fungal cultures from bronchioloalveolar lavage (BAL) were negative. BAL cytology was negative for eosinophilia and showed only mild lymphocytosis of 26%. Infectious pneumonitis and hypersensitivity pneumonitis were, therefore, ruled out. 

**Figure 3:**
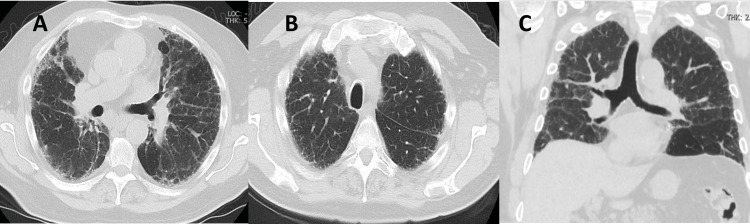
CT scan showing resolution of ground glass opacities. A, transverse view of lung (proximal); B, transverse view of lung (distal); C, sagittal view of lung

Over the course of the hospital stay, the patient continued to have increasing oxygen requirements and worsening hypoxic respiratory failure due to which the decision was made to initiate emperic steroids with IV salumederol 40 mg twice a day. Within 72 h, the patient reported feeling back to baseline and oxygen requirements improved. He was then started on a steroid taper with prednisone 100 mg daily for 3 days, followed by a taper to 60 mg daily for 7 days, then 40 mg daily for 7 days, and then maintenance on 30 mg daily. The patient was discharged to home. Repeat chest X-ray (Figure 1B) four weeks later showed overall improvement in reticular abnormalities that were seen on admission. A repeat CT scan 3 months out, showed improvement in ground glass opacities (Figure 3A-C). The patient was gradually tapered off prednisone within 3 months and infliximab was held indefinitely. The patient did not have any further relapses in the next 4 months.

## Discussion

Infliximab is a weight based, intravenously administered chimeric immunoglobulin G1 antibody that targets the tumor necrosis factor (TNF) alpha cytokine and is used for both induction and maintenance therapy for IBD. Infliximab was the first monoclonal antibody to be approved for the treatment of pediatric and adult patients with moderately to severely active CD and UC [2]. It was first approved by the Food and Drug Association in 1998 as a treatment for CD [3] and since then, its use has been expanded to UC as well as other chronic inflammatory diseases such as rheumatoid arthritis, psoriatic arthritis, and ankylosing spondylitis. Infliximab has been shown to induce and maintain both clinical remission and mucosal healing in pediatric and adult patients with IBD. Infliximab targets both soluble and transmembrane TNF-alpha, a potent proinflammatory cytokine that plays a role in dysregulation of the mucosal immune response [2].

One of the common adverse effects that plagues the use of infliximab is the development of autoantibodies against the drug. This in return leads to a loss of its efficacy. A 2017 study showed that in adult patients with CD, the incidence of loss of response to infliximab ranged from 8% to 71% [4]. Similarly, loss of response has been documented in pediatric patients with IBD with estimates of 20%-50% of pediatric patients with IBD who initially responded to induction therapy with infliximab, lose the response within 1 year [2]. Other common adverse effects that are well documented include reactivation of latent infections such as tuberculosis and hepatitis B, psoriatic cutaneous lesions, infusions-related reactions, and hepatotoxicity. However, data have also surfaced regarding ILD as an adverse event associated with the use of TNF alpha inhibitors and this association is increasingly being reported since 2002 [5]. This association, however, appears to be more commonly seen in patients with prior history of ILD or who are being treated with infliximab for rheumatological conditions such as rheumatoid arthritis or psoriasis [6].

In their 2010 study Perez-Alvarez et al. looked at cases of new onset or exacerbation of ILD in patients between January 1990 and March 2010. They found 122 cases of ILD in which 58 cases were associated with etanercept and 56 cases were associated with infliximab. They observed that evidence of ILD appeared approximately 26 weeks after initiation of the biologic agent. Out of the 26 cases that underwent lung biopsy, six were identified as NSIP [6]. Similarly in 2013, Kakavas et al. reported a case of ILD associated with infliximab just 3 weeks after initiation of therapy for psoriasis which resolved following discontinuation of treatment and a short course of steroid treatment [7]. More recently in 2022, Sawal and Skalski reported a case of a 65-year-old with rheumatoid arthritis who was started on infliximab therapy and developed ILD. This too resolved within 1 month of discontinuation of the drug [8].

While less frequent, there also have been reports documenting ILD in patients treated with infliximab for IBD. To our knowledge, the first report was published by Weatherhead et al. in which a patient developed ILD after a single infusion of infliximab for CD [9]. The second report was published by Wiener et al. and in this case, the patient developed ILD after five infusions [10]. In both cases, symptoms resolved after infliximab discontinuation and a short steroid course. In 2012 Wen et al. reported a case of NSIP after the second infusion of infliximab in a patient who was being treated for UC [11]. Finally in their 2013 study, Caccaro et al. reported the case of a patient who was treated with infliximab for Crohn’s disease and developed NSIP after the fifth infusion with resolution of imaging findings 8 weeks after drug discontinuation and steroid treatment [12].

The pathogenesis of this phenomenon is not clearly understood. It is hypothesized that infliximab-induced ILD may be a form of CD8 T-cell mediated hypersensitivity reaction given that there have been reports of cutaneous delayed-type hypersensitivity reactions to infliximab [11]. In our patient, we found that the BAL was positive for 26% lymphocytes which may not be sufficient to generate this adverse reaction. In their study, Torres et al. described a perivascular lymphocytic infiltrate composed predominantly of CD8 T cells with paucity of eosinophils in skin biopsies taken from patients receiving infliximab, who developed maculopapular exanthemas [13].

## Conclusions

Based on our literature review, ILD associated with infliximab use in IBD generally presents early after a few infusions. This is contrary to our patient’s case who had been under treatment with infliximab for more than 24 months. The type of ILD can be variable and NSIP is one example that has been consistently reported same as our patient. Fortunately, in all reports, it appears that this adverse effect may respond well to a steroid course and discontinuation of the drug with resolution of physical damage to the lung parenchyma as evidenced by the imaging studies post drug discontinuation. In our patient, we anticipate there will be a full resolution of lung changes with time. We, therefore, present this case in order to raise awareness regarding this poorly understood phenomenon and advise clinicians using infliximab to maintain a high index of suspicion for reversible ILD and recommend early discontinuation of drug if there is concern for a pulmonary process in the setting of infliximab use.
